# A Review of Piezoelectric Vibration Energy Harvesting with Magnetic Coupling Based on Different Structural Characteristics

**DOI:** 10.3390/mi12040436

**Published:** 2021-04-14

**Authors:** Junxiang Jiang, Shaogang Liu, Lifeng Feng, Dan Zhao

**Affiliations:** 1 College of Mechanical and Electrical Engineering, Harbin Engineering University, Harbin 150001, China; jiangjunxiang2014@163.com (J.J.); heuzhaodan@outlook.com (D.Z.); 2 School of Mechanical and Civil Engineering, Jilin Agricultural Science and Technology University, Jilin 132101, China; 3 Beijing Institute of Precision Mechatronics and Controls, CALT, Beijing 100076, China; feng_lifeng@yeah.net

**Keywords:** piezoelectric energy harvester, vibration, magnetic coupling, energy-conversion device

## Abstract

Piezoelectric vibration energy harvesting technologies have attracted a lot of attention in recent decades, and the harvesters have been applied successfully in various fields, such as buildings, biomechanical and human motions. One important challenge is that the narrow frequency bandwidth of linear energy harvesting is inadequate to adapt the ambient vibrations, which are often random and broadband. Therefore, researchers have concentrated on developing efficient energy harvesters to realize broadband energy harvesting and improve energy-harvesting efficiency. Particularly, among these approaches, different types of energy harvesters adopting magnetic force have been designed with nonlinear characteristics for effective energy harvesting. This paper aims to review the main piezoelectric vibration energy harvesting technologies with magnetic coupling, and determine the potential benefits of magnetic force on energy-harvesting techniques. They are classified into five categories according to their different structural characteristics: monostable, bistable, multistable, magnetic plucking, and hybrid piezoelectric–electromagnetic energy harvesters. The operating principles and representative designs of each type are provided. Finally, a summary of practical applications is also shown. This review contributes to the widespread understanding of the role of magnetic force on piezoelectric vibration energy harvesting. It also provides a meaningful perspective on designing piezoelectric harvesters for improving energy-harvesting efficiency.

## 1. Introduction

Vibration sources can be found in different environments ranging from human motion, bridges, industrial equipment, and vehicle vibration to household appliances, etc. Energy-harvesting techniques that convert ambient vibrations into electrical energy for charging self-powered electronic devices have attracted a lot of attention over the past decade [1,2,3,4]. For vibration-based energy harvesters, there are mainly four different methods that are most extensively used by scholars in the process of research and application, including piezoelectric [5,6,7,8], electrostatic [9,10,11], electromagnetic [12,13,14], and triboelectric transductions [15,16,17]. Piezoelectric energy harvesting is popular due to the merits of high energy density, easy miniaturization, simple structure, and easy application characteristics of piezoelectric materials. In many real applications, ambient vibration frequencies are often random and broadband, and the design of energy harvesters needs to adapt to these vibration characteristics. The key issue is how to make the frequency of a harvester match the external frequency with a wider frequency bandwidth for applications.

In general, the performance of linear piezoelectric energy harvesting is limited to a very narrow frequency bandwidth [18,19,20]. So, many methods have been proposed to realize broadband energy harvesting and improve energy-harvesting efficiency, including multimodal [21,22], frequency tuning [23,24,25], frequency-up [26,27,28,29], nonlinear approaches [30,31,32,33,34], etc. In addition, the design of new piezoelectric materials, such as organic–inorganic nanocomposites, is a promising strategy for improving the energy-conversion efficiencies. Many methods for the improvement of the piezoelectric response of polymeric materials and scaffolds have been developed by materials scientists [35]. Among various piezoelectric energy-harvesting structures, permanent magnets are often attached to associated structures to improve structural properties. It is easy to design a nonlinear energy harvester with magnetic coupling, with characteristics such as magnetic frequency tuning [36,37,38], magnetic frequency up-conversion [39,40,41,42,43], monostable [44,45], bistable [46,47,48], and multistable [49,50,51,52,53]. Bouhedma et al. presented a novel vibration-based piezoelectric energy harvester with magnetic frequency-tuning [54]. Compared with a single-frequency harvester, the frequency of this harvester can be tuned by up to 18% both up and down. The presented approach combining multiresonance and frequency tunability acquired a larger operative bandwidth. Yang et al. presented a press-button-type piezoelectric energy harvester, in which the frequency upconversion concept was adopted to generate high power density [55]. The converted energy was magnified by an impact between magnets. Cottone and Gammaitoni proposed a bistable oscillator for a generic wide-spectrum vibration [56]. The dynamics of this harvester could be controlled with the magnetic force and polarities of the added magnets. There was a range of distances between magnets, and the voltage reached a maximum value under random excitations. The output power was improved between 400 and 600% compared to the linear one in experiments. Tang et al. investigated the functionality of vibration energy harvesters using magnets. Both monostable and bistable configurations introduced by magnets were discussed with various excitation levels to improve the performance of vibration energy harvesting [57]. Zhou et al. investigated the dynamic characteristics of broadband tristable energy harvesters with magnetic coupling [58]. Compared with the bistable state, the results showed that this harvester was easily excited to pass potential wells for producing high energy output over a wider range of frequencies. Wang et al. proposed a wideband quin-stable energy harvester with magnetic nonlinearity [59].

In addition, magnets are usually arranged to pluck the piezoelectric beam with a repelling or an attractive configuration [60,61,62,63]. Relative movement of the magnet in relation to the coil causes a variation magnetic flux inside the coil, and the voltage induced in the coil can be obtained by Faraday’s law. This hybrid approach increases the power generation capability of the energy harvester due to the combinational piezoelectric and electromagnetic operation under the same external excitation [64,65,66].

Moreover, a new generation of devices can be developed using multiferroic materials and structures based on magnetoelectric effects. Magnetoelectric effects in ferromagnetic–ferroelectric layered composites arise due to magnetostriction and the piezoelectric effect in the ferroic phases, and these structures are driven by mechanical strain. Compared with the mechanical method of piezotronics, such a magnetic-field-based method via magnetoelectric coupling effects has some important advantages because it is remote and contactless. Multiferroic composites have the potential for applications in sensors, autonomous energy sources, electrically controlled microwave devices, and memory elements [67,68,69,70,71].

There are some published reviews about piezoelectric energy harvesting, and yet most of them are primarily focused on topics including biomedicine [72,73,74,75,76,77], micro-electro-mechanical-systems (MEMS) [78,79,80,81,82], nanogenerators [83,84,85], materials [86,87,88,89,90], and variety energy harvesting [81,82,83,84,85,86,87,88,89,90,91,92,93,94]. However, this review focuses on the piezoelectric vibration energy harvesters with magnetic coupling. This review aims to classify five piezoelectric vibration energy harvesting technologies with magnetic coupling and determine the potential benefits of magnetic force on energy-harvesting techniques. We mainly focus on vibration energy sources in mechanical vibrations and human activities, and did not include flow energy sources like wind, sound, or ocean energy in this paper. In the analysis of different designs of energy-harvesting system, it is favorable to develop a harvester including magnets to enhance the efficiency of energy harvesting from vibrations to convert them to electrical energy. This paper will also discuss the applications of different energy-harvesting devices with magnetic coupling. The detailed introduction and application of the harvesters are discussed in the following sections.

A comprehensive review of recent studies on piezoelectric vibration energy harvesting is presented. Then, it is shown that magnetic force plays an important role in energy harvesting. This review is organized as follows. The second to sixth sections illustrates detailed descriptions of the five types of piezoelectric vibration energy harvesting technologies with magnetic coupling, including monostable, bistable, multistable, magnetic plucking, and hybrid piezoelectric–electromagnetic methods. Working principles and representative designs are also displayed in these sections. Then, the practical applications of the harvesters are generalized in the seventh section. The last section summarizes this review and discusses the outlook for piezoelectric energy harvesting.

## 2. Monostable Energy Harvesters

### 2.1. Working Mechanism

Wider bandwidth for energy harvesting can be achieved by proper sweep or by magnet spacing, especially for the hardening or softening configurations, according to the forced Duffing equation. A monostable nonlinear energy harvester can be implemented using magnetic repulsion and magnetic attraction [95,96,97,98]. It is divided into the magnet parallel to the piezoelectric beam and perpendicular to the piezoelectric beam, based on the arrangement of magnets and piezoelectric beams.

The dynamic behavior for vibration energy harvesters can be described by a model, known as the Duffing oscillator. To gain a more general understanding, a physics-based model consisting of a lumped-parameter mechanical oscillator coupled with an electromechanical coupling was established [48,99,100].

The nondimensional equations of motion can be expressed as Equations (1) and (2).

(1) $$\ddot{x}+2\zeta \dot{x}+\frac{dU}{dx}+\kappa^{2}y=-{\ddot{x}}_{b}$$

(2) $$\dot{y}+\alpha y=\dot{x}$$

where

(3) $$\frac{dU}{dx}=(1-r)x+\delta x^{3}$$

where the second derivative of *x_b_* is the base acceleration, *x* represents the relative displacement of the mass, *y* is the electric quantity representing the induced voltage in capacitive harvesters, *ζ* is the mechanical damping ratio, *κ* is a linear dimensionless electromechanical coupling coefficient, *α* is the ratio between the mechanical and electrical time constants of the harvester, *U* represents the potential energy of the mechanical subsystem that leads to cubic nonlinearities in the mechanical oscillator, *r* is introduced to permit variations in the linear stiffness around its nominal value, and *δ* is the coefficient of the cubic nonlinearity.

The form of Equation (3) divides energy harvesters into three major categories based on the shape of their potential energy function. When *δ* = 0 and *r* < 1, the system is a linear harvester. When *δ* > 0 and *r* ≤ 1, the system is a monostable hardening type. When *δ* < 0 and *r* ≤ 1, the system is a monostable softening type. When *δ* > 0 and *r* > 1, the system is a nonlinear bistable one. The potential function of the harvester has two potential wells separated by a potential barrier. For the monostable harvesters, the frequency-response curves bend which can make the bandwidth of energy harvesters wider.

### 2.2. Magnets Parallel to the Piezoelectric Beam

**Figure 1:**
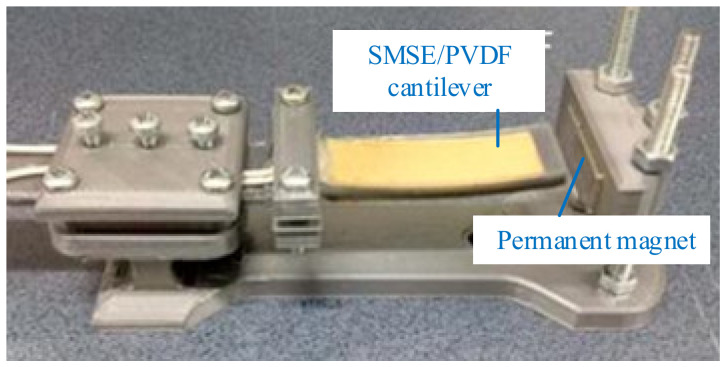
A soft magneto-sensitive elastomer (SMSE) harvester (reproduced with permission from [104]; published by IOP, 2018).

For this kind of monostable-type piezoelectric energy harvester, the magnets are arranged in parallel with the piezoelectric beam [101,102,103]. Yang et al. developed a broadband vibration energy harvester based on a soft magneto-sensitive elastomer (SMSE), which was attached a polyvinylidene fluoride (PVDF) to convert vibration energy into electricity [104] (Figure 1). The device exhibited a strong softening effect when it was subjected to the magnetic field. When the load resistance was 4.7 MΩ and the SMSE thicknesses was 5 mm, the corresponding power was 0.11 μW over a wide frequency range at an acceleration level of 0.3 g. Deng and Wang reported an input-dependent performance study of a nonlinear piezoelectric energy harvester with introduced magnetic interaction [105]. The performances of this harvester with two external magnet arrays (I and II) were compared. The harvester of Array II, which had a symmetric magnetic force, yielded better voltage output under a frequency sweep test. Compared with the linear one, the output voltage improved 104.5% and the bandwidth was 7 Hz under the excitation of 0.334 g.

In addition, a two degrees of freedom (2-DOF) system has been studied by scholars. Krishnasamy et al. proposed a 2-DOF harvester to combine multimodal energy harvesting with the nonlinear vibration technique [106,107,108]. By properly choosing the structural parameters and the distance between the magnets, two resonant response peaks could be tuned to be close enough and with adequate amplitude. Upadrashta and Yang proposed an array of nonlinear piezomagnetoelastic energy harvesters for scavenging electrical energy from broadband vibrations with low amplitudes [109]. The dynamic responses of two harvesters, one with an attractive configuration and the other one with a repulsive configuration, were combined to achieve a bandwidth of 3.3 Hz at a power level of 100 μW under harmonic excitation of 2 m/s^2^.

**Figure 2:**
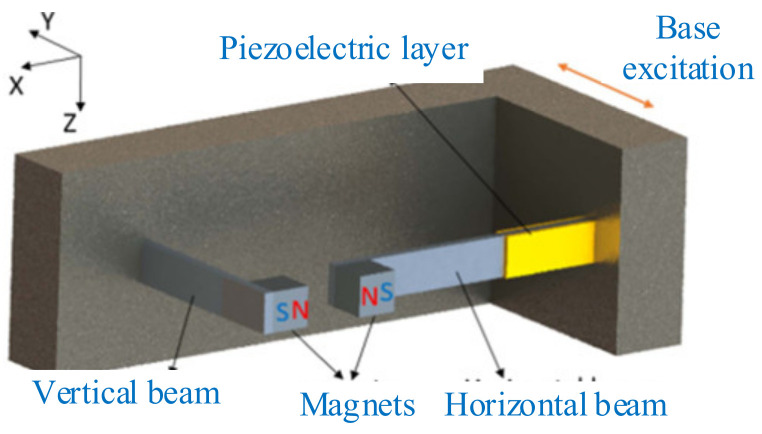
An internal resonance harvester formed by two beams (reproduced with permission from [114]; published by IOP, (2017).

The harvester may exhibit internal resonance, when the linear natural frequencies are commensurable or nearly commensurable [110,111]. To improve the frequency bandwidth, an L-shaped 2:1 internal piezoelectric harvester energy with frequency tuning magnets was proposed by Chen et al. [112]. Xiong et al. developed a 2:1 internal resonance harvester with magnetic interaction and an auxiliary oscillator for enhancing broadband vibration energy harvesting [113]. Yang and Towfighian proposed a 2-DOF harvester consisting of two resonators, each with a permanent magnet on its tip. Internal resonance could be triggered by adjusting the distance between two magnets [114] (Figure 2).

### 2.3. Magnets Perpendicular to the Piezoelectric Beam

**Figure 3:**
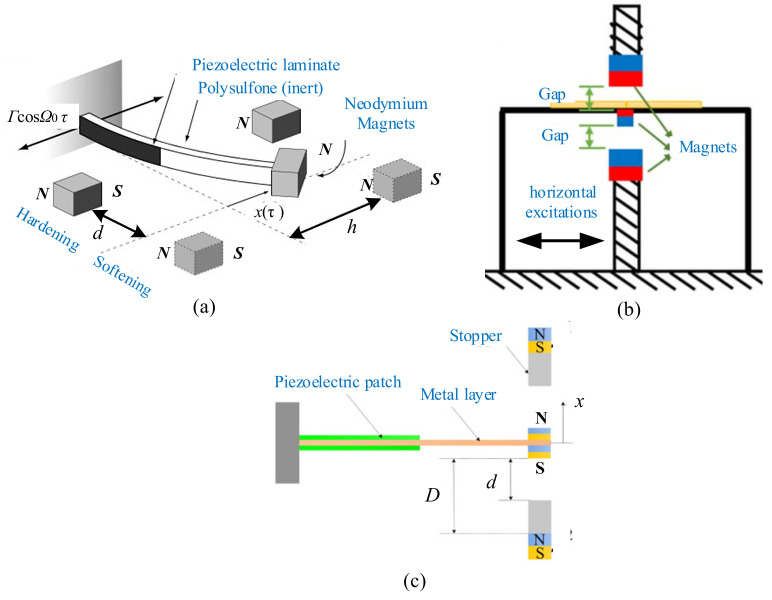
The monostable harvesters with the magnets perpendicular to the piezoelectric beam. (**a**) Magnetopiezoelastic energy harvester (reproduced with permission from [122]; published by American Institute of Physics, (2009). (**b**) U-shaped bidirectional harvester (reproduced with permission from [123]; published by IOP, 2018). (**c**) Monostable energy harvester (reproduced with permission from [125]; published by AIP, 2018).

**Figure 4:**
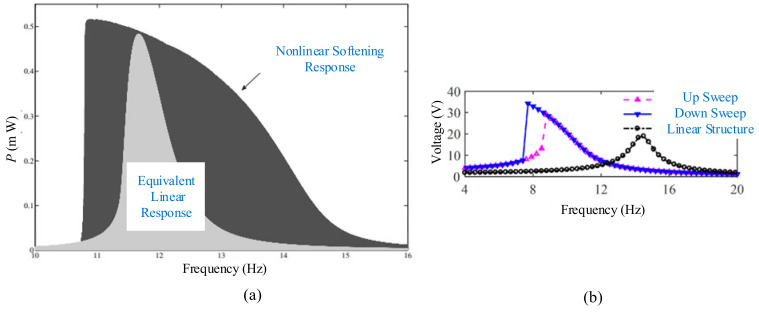
Nonlinear and linear energy-harvest responses. (**a**) The research results by Stanton et al. (reproduced with permission from [122]; published by American Institute of Physics, 2009). (**b**) The research results by Fan et al. (reproduced with permission from [125]; published by AIP, 2018).

For this kind of monostable type piezoelectric energy harvester, the magnets are perpendicular to the piezoelectric beam [115,116,117,118,119,120,121]. Stanton et al. modeled and experimentally validated a nonlinear energy harvester capable of bidirectional hysteresis [122] (Figure 3a). By tuning nonlinear magnetic interactions around the end mass, hardening or softening responses occurred, increasing the bandwidth of the device in either direction. For the same exciting amplitude of 0.3 g, the exciting frequency was linearly decreased for both the nonlinear and linear configurations. Figure 4a shows that the monostable harvester has a wider bandwidth in comparison to the linear beam. Shih and Su proposed a magnet-induced nonlinear U-shaped bidirectional piezoelectric harvester to overcome narrow bandwidth and unidirectional acquisition [123] (Figure 3b). A strong hardening effect could be observed in horizontal excitations when the magnet gap was small.

A combination of monostable, hybrid methods was applied to structural design. Fan et al. presented a monostable harvester for achieving enhanced energy from low-level excitations by introducing symmetric magnetic attraction and a pair of stoppers to confine the maximum deflection of the beam [124,125] (Figure 3c). The experimental results showed that the proposed device outperformed its linear counterpart in terms of output power (253% increase) and operating bandwidth (54% increase) under a sinusoidal vibration with an acceleration of 3 m/s^2^. The monostable harvester exhibited not only a much wider bandwidth, but also a significantly larger peak voltage than the linear one when *D* was 22 mm and *d* was 8.7 mm, as shown in Figure 4b. Zhang et al. presented a magnetically levitated harvester with a 2-DOF cantilever to performance dependence on initial free-end levitation position [126]. It was found that the case above the horizontal orientation had a stronger impact on the performance than that below the horizontal orientation. Moreover, the former brought a more significant influence on the second resonance frequency than the first one. The dynamic performances were affected by the initial free-end levitation position.

### 2.4. Characteristic Analysis of the Monostable Harvesters

The monostable energy harvester is constructed in a variety of flexible ways, including different magnet arrangements, attraction or repulsion, number of beams, etc. Table 1 illustrates the performances of some existing monostable type piezoelectric harvesters in recent years. It can be observed clearly that both types of magnet arrangement (parallel and perpendicular) are widely used, but there are some exceptions. Yang and Towfighian introduced magnetic nonlinearity to a 2-DOF system by combining the softening and hardening behaviors [103]. Two piezoelectric cantilevers with magnets were used. One cantilever was in the horizontal plane, but the other was in the vertical direction. The frequency bandwidth of this harvester was widened by the introduced magnetic nonlinearity, which decreased the threshold excitation value.

The nonlinear magnetic force is complex and it depends on many variables, such as spacing distance between the magnets, polarity of magnets, and their dimensions. Abdelmoula et al. performed a comparative study on a broadband piezoelectric energy harvester with single and dual magnetic forces [95]. The results showed that hardening behaviors took place in the dual attractive magnets, but softening behaviors took place in the single-magnet design. Rui et al. studied four different harvester modes containing low/high frequency beams with attraction/repulsion [97]. A low beam with repulsion mode was selected due to its high output power in a wide operating frequency range. In addition, the internal resonance phenomenon can occur due to a nonlinear configuration using magnets.

## 3. Bistable Energy Harvesters

### 3.1. Working Mechanism

Compared to the monostable system, the main advantage of the bistable system lies in the fact that the interwell motion can lead to a large deformation, and then generate greater output power. However, the dynamics of the bistable configuration are more complicated than those of the monostable one. Small-amplitude oscillations in one potential well, large-amplitude oscillations crossing two potential wells, or even chaotic responses may appear, depending on the base excitation level and the initial condition. Magnetic attraction or magnetic repulsion can induce the bistability [127,128,129,130,131,132,133,134,135].

**Figure 5:**
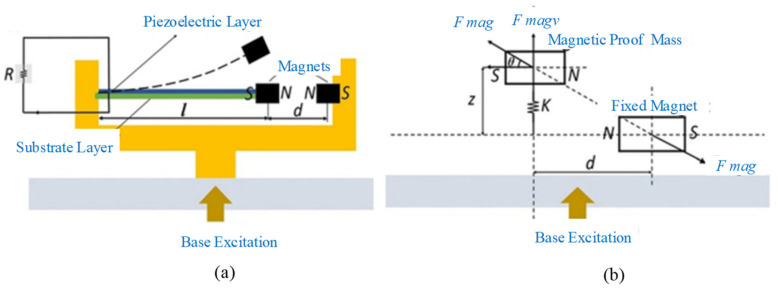
(**a**) Schematic of piezoelectric cantilever beam with magnetic proof mass (reproduced with permission from [136]; published by Wiley-VCH Verlag GmbH & Co. KGaA, Weinheim, 2015. (**b**) Free-body diagram of magnets (reproduced with permission from [136]; published by Wiley-VCH Verlag GmbH & Co. KGaA, Weinheim, 2015.

A typical bistable nonlinear piezoelectric energy harvester is shown in Figure 5a [136]. Two magnets were used to generate a repulsive force. The magnetic force is approximated as a cubic function that makes the system monostable or bistable, depending on the distance between the magnetic proof mass and fixed magnet. The horizontal component of the magnetic force is neglected due to the small tip displacement of the beam, and the vertical component can be obtained by Equation (4), as shown in Figure 5b.

(4) $$F_{magv}=F_{mag}\sin\theta =F_{mag}\frac{\tan\theta }{\sqrt{1+\tan^{2}\theta }}=\frac{F_{mag}}{d}\frac{z}{\sqrt{1+{\left(\frac{z}{d}\right)}^{2}}}$$

where *F*_mag_ is the magnetic force, *F*_magv_ is the vertical component of magnetic force, *d* is the distance between the two magnets, and *z* is the relative displacement of the magnetic proof mass. Equation (4) is expanded using the Taylor’s series method, and only the first two terms are considered because the contribution of the higher terms is negligible.

(5) $$F_{magv}≅\frac{F_{mag}z}{d}-\frac{F_{mag}z^{3}}{2d^{3}}$$

where

$$\frac{F_{mag}}{d}=K_{1},\;\frac{F_{mag}}{2d^{3}}=K_{2}.$$

When the beam vibrates from the equilibrium position, a restoring force due to the linear stiffness of the beam acts downward, and a counteracting restoring magnetic force acts in the upward direction. The net nonlinear force *F*(*z*) is:

(6) $$F(z)=(K-K_{1})z+K_{2}z^{3}$$

The potential energy function of the piezoelectric system is given as:

(7) $$U(z)=\frac{1}{2}(K-K_{1})z^{2}+\frac{1}{4}K_{2}z^{4}$$

where *K* = linear stiffness.

According to Equation (3), for *K* − *K*_1_ < 0 and *K*_2_ > 0, a bistable configuration is achieved. Bistable oscillators have a unique double-well restoring force potential [46]. The bistable devices may exhibit intrawell, chaotic, or interwell dynamics, depending on the initial conditions and exciting magnitude and frequency. The periodic interwell dynamics, alternatively high energy orbits or snap-through, have been recognized to dramatically improve energy-harvesting performances. However, the required velocity of the device is much greater than intrawell or chaotic dynamics.

**Figure 6:**
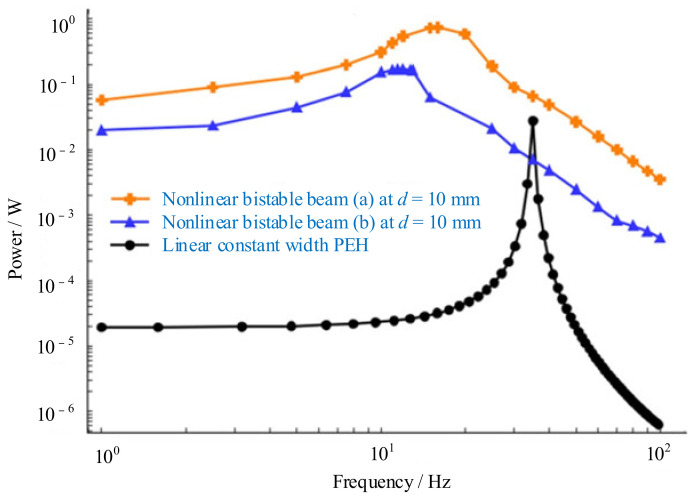
Power generated by bistable and linear harvesters at *d* = 10 mm (reproduced with permission from [136]; published by Wiley-VCH Verlag GmbH & Co. KGaA, Weinheim, 2015.

The generated average power during harmonic base excitation of bistable beams and the linear rectangular one (without considering nonlinearity in stiffness) were plotted in Figure 6. It showed bistable piezoelectric beams could generate substantially higher power values over a wide range of vibration frequencies compared with the linear one [136].

### 3.2. Magnetic-Repulsion Bistability

**Figure 7:**
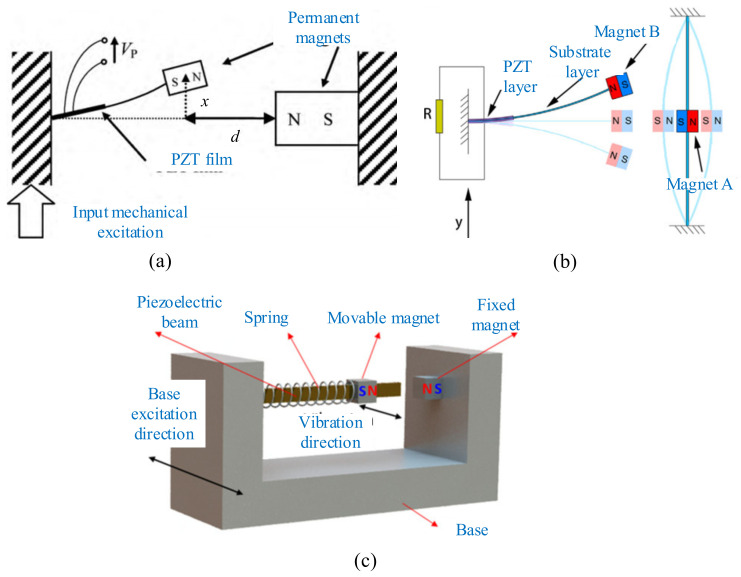
The bistable harvesters with magnetic repulsion. (**a**) Bistable system (reproduced with permission from [143]; published by Elsevier B.V., 2010). (**b**) Nonlinear flexible harvester (reproduced with permission from [145]; published by Elsevier, 2018). (**c**) Hybrid nonlinear harvester (reproduced with permission from [146]; published by Elsevier, 2016).

Most vibration energy harvesters use a single degree-of-freedom system [136,137,138,139,140,141,142]. The typical bistable structure consisted of a piezoelectric beam converter coupled to permanent magnets [143], as shown in Figure 7a. The magnets were mounted with opposed polarities, so that the resulting magnetic force was repulsive. Under proper conditions, the structure bounced between two stable states in response to external excitation, which significantly improved energy harvesting from wide-spectrum vibrations.

When vibration excitation intensity is insufficient, bistable energy-harvesting systems are not able to realize reciprocating transition oscillations between two wells. To overcome this defect, a combination of bistability and flextensional mechanisms was applied to structural design. Leng et al. conceived an elastic-support model to study the performance of nonlinear piezoelectric vibration energy converters under variable-intensity excitation conditions [144]. The results showed that with the magnet interval and the spring’s elastic stiffness unchanged, the elastic-support system had greater power-output performance than a rigid-support system under low-intensity filtered Gaussian noises. Zhou et al. developed a nonlinear flexible bistable energy harvester that comprised a piezoelectric cantilever beam with a tip magnet and a clamped-clamped beam with a midmagnet [145] (Figure 7b). Owing to the variable repulsive magnetic force, the harvester could realize snap-through more easily and create large output voltages. Yang et al. combined bistability and internal resonance effects to broaden the frequency bandwidth of a harvester. The hybrid harvester consisted of a piezoelectric cantilever beam carrying a movable magnet and a fixed magnet [146] (Figure 7c). The spring on the beam allowed the movable magnet to slide along the beam. Compared with bistable energy harvesters with two fixed magnets that had only one branch, the bandwidth of the new design was two times larger.

Several multi-degree-of-freedom approaches have been proposed to increase the frequency bandwidth [147,148,149,150,151]. Gao et al. developed a bistable dual-piezoelectric-cantilever harvester to achieve optimal broadband energy harvesting under varying-intensity realistic circumstances [152].

### 3.3. Magnetic-Attraction Bistability

**Figure 8:**
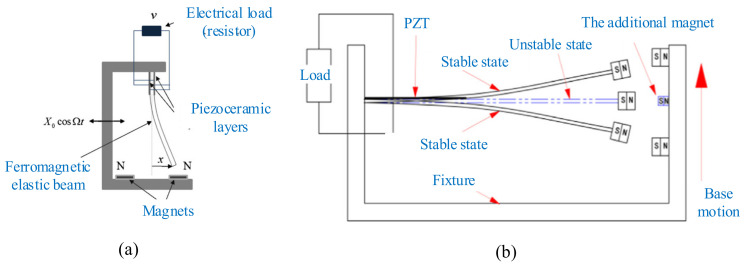
The bistable harvesters with magnetic attraction. (**a**) The piezomagnetoelastic energy harvester (reproduced with permission from [157]; published by Elsevier, 2010). (**b**) An improved bistable energy harvester (reproduced with permission from [158]; published by Elsevier, 2016).

The use of magnetic attraction to induce bistability in energy harvesting was studied by some scholars [153,154]. Erturk et al. investigated the use of magnetic attraction to induce the bistability of a piezomagnetoelastic beam. The device consisted of a ferromagnetic cantilevered beam with two permanent magnets located symmetrically near the free end, and it was subjected to base excitation [155,156,157] (Figure 8a). The configuration could generate power an order of magnitude larger compared to commonly employed piezoelastic configurations over a range of frequencies.

Generally, the deep potential wells can bring about the large vibration amplitude. However, the deep wells will create a high barrier. When the harvester is driven by weak excitation, its harvesting efficiency will decrease due to the intrawell motion. Lan et al. developed an improved bistable energy harvester to prompt the ability of harvesting energy [158] (Figure 8b). An additional small magnet was added to the classical bistable harvester, and its magnetic force could pull down the barrier between potential wells. Thus, this device could produce a high output voltage even with weak excitation.

### 3.4. Characteristic Analysis of the Bistable Harvesters

The structural characteristics of some existing bistable-type piezoelectric harvesters are listed in Table 2. It can be found that the bistable structures using magnetic repulsion are widely used compared with magnetic attraction. Magnetic repulsion can reduce the resonance frequency of the structure, which makes it easier to harvest the low-frequency vibration energy. In addition to cantilever, the shape of piezoelectric beams can be arc-shaped, L-shaped, or buckled. Zou et al. proposed a compressive-mode wideband vibration energy harvester that combined the advantages of the bistable oscillator and the flextensional actuator [135]. The flextensional actuator was composed of one PZT layer and two raised metal layers bonded to both sides of the PZT layer. With one flextensional actuator, the instantaneous power was up to 31 μW, across a load resistance of 390 kΩ under a base excitation of 0.8 g. Moreover, the number of piezoelectric beams could be one, two, or three.

Most of the literature listed studied the excitation direction when perpendicular to the piezoelectric beam. However, Pan et al. proposed an improved inverted beam harvester, and the excitation was applied parallel with the beam in the vertical direction [139]. The additional magnets of this inverted piezoelectric beam could tailor the potential energy, allowing this harvester to undergo snap-through under weak excitation. In addition, Zou et al. presented a magnetically coupled vibration energy harvester using two inverted piezoelectric beams for rotary-motion applications [159]. The high voltages of the first and second resonant points could be obtained at rotating speeds of 420 r/min and 550 r/min, and the corresponding average output powers were 564 μW and 535.3 μW, respectively.

## 4. Multistable Energy Harvesters

### 4.1. Working Mechanism

Many studies indicated that bistable energy harvesters showed advantages both in frequency bandwidth and average power density. However, due to the barrier in the potential function, the large-amplitude interwell oscillation does not occur all the time, because the intensity of the vibration sources in nature is uncertain. To solve this problem, tristable [160,161,162,163,164,165,166,167], quadstable [168,169,170,171] and even pentastable [172,173,174] configurations were investigated recently. With more potential wells, the potential energy can be distributed more uniformly, and the barriers between the potential wells can be lower, even though the barriers between the potential wells still exist.

**Figure 9:**
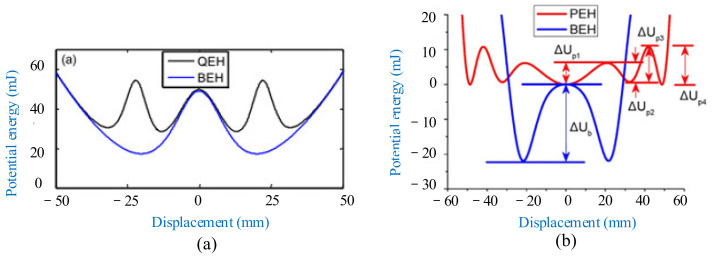
Potential energy shapes for: (**a**) Quadstable (QEH) and bistable (BEH) (reproduced with permission from [169]; published by Elsevier, 2016); and (**b**) pentastable (PEH) and bistable (BEH) (reproduced with permission from [174]; published by Elsevier B.V., 2017).

The total potential energy of the harvester can be broken up into two components: the elastic potential energy and the magnetic one. The potential energy shapes for quadstable and bistable are plotted in Figure 9a [169]. The potential energy shapes for pentastable and bistable are plotted in Figure 9b [174]. The comparison showed that multistable harvesters had shallower potential wells, and made the distance between the outermost two wells larger. The decrease in depth of the potential well implied that the response of the multistable harvester required quite low energy to realize jumping between the potential wells. Jumping between the nonadjacent wells could create larger amplitudes and generated more power accordingly.

**Figure 10:**
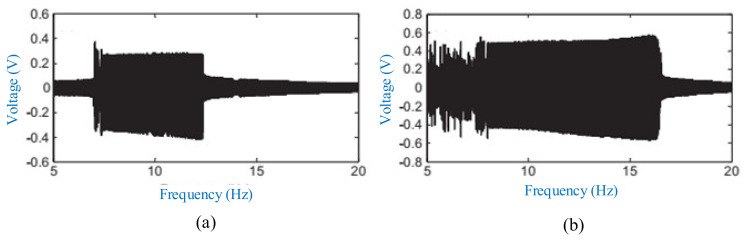
Tested voltages **of energy harvesters** under frequency-sweeping excitation. (**a**) Bistable; (**b**) quadstable (reproduced with permission from [169]; published by Elsevier, 2016).

Figure 10 shows the bistable and quadstable experimental responses under a frequency-sweeping excitation. The quadstable harvesters own a smaller threshold and a wider range of frequencies for occurrence of snap-though than the bistable harvesters. Thus, the multistable energy harvesters show an advantage in scavenging vibration energy under certain conditions.

### 4.2. The Tristable Harvesters

The common type tristable structure consisted of a piezoelectric beam with two external permanent magnets [175]. Magnet A on the tip of the cantilever beam was the opposite polarity to magnets B and C, so that the resulting magnetic force was repulsive. This structure had three equilibrium positions from Table 1, Table 2 and Table 3 in the static state. From the potential energy figures, this harvester had four transitions among monostable, bistable and tristable.

**Figure 11:**
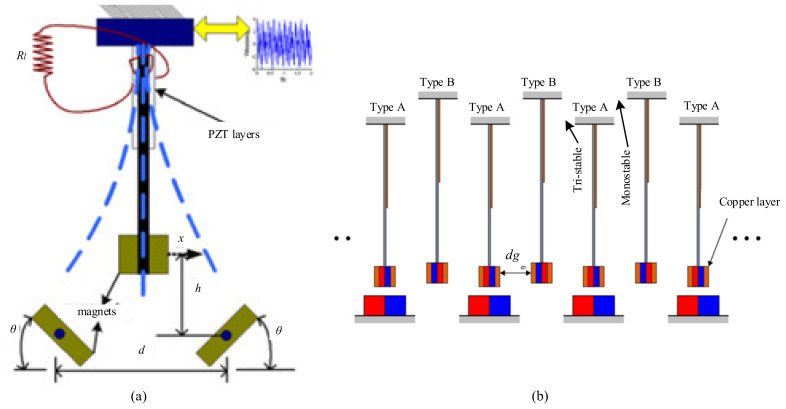
The tristable harvesters. (**a**) A tristable nonlinear oscillator (reproduced with permission from [161]; published by AIP, 2015). (**b**) A nonlinear multistable energy-harvester array (reproduced with permission from [165]; published by Elsevier, 2018).

Oumbé Tékam et al. analyzed a tristable energy harvester with fractional order viscoelastic material. The harvester consisted of a piezoelectric cantilever with three magnets placed on the base. The Melnikov theory was used to understand the effects of the physical property of this flexible material [176]. Cao et al. investigated a tristable nonlinear harvester for exploring the performance with different potential well functions under harmonic excitations. The results showed that there was an optimum shallower potential well function that would enhance the effective frequency width under low-frequency excitation [161] (Figure 11a). Lai et al. investigated a nonlinear multistable energy-harvester array combining a tri-stable and a monostable construction [165] (Figure 11b). These harvesters were arranged alternately, and the tip magnets generated a nonlinear repulsive force. By utilizing the interaction between harvesters, this structure had the advantages of higher harvesting efficiency and wider operating bandwidth under low-amplitude (<3 m/s^2^) and low-frequency (<20 Hz) vibrations.

### 4.3. The Quadstable Harvesters

**Figure 12:**
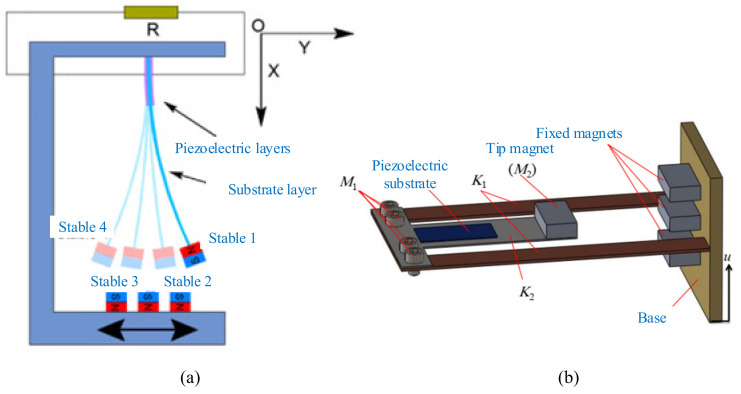
Some existing quadstable harvesters. (**a**) A quadstable harvester with one degree of freedom (reproduced with permission from [170]; published by Elsevier, 2018). (**b**) A quad-stable harvester with two degrees of freedom (2-DOF) (reproduced with permission from [171]; published by MDPI, 2019).

To improve the energy-conversion ability, a quadstable energy harvester was explored by Zhou et al. Four stable equilibrium positions could be realized in the static state by adjusting the positions and distances between the tip and the fixed magnets [170] (Figure 12a). The research results showed that this harvester exhibited better performances on dynamic response and output voltage than the bistable one.

Two degrees of freedom approaches had been proposed to increase the frequency bandwidth. Abdelhameed et al. developed a 2-DOF quadstable cut-out vibration energy harvester. The tip magnet on the end of the inner beam was repelled by three permanent magnets fixed to the base. This harvester showed a higher figure of merit (167%) than that of the bistable one [171] (Figure 12b). In addition to the tri-stable and quad-stable harvesters, pentastable energy harvesting systems also have been proposed.

### 4.4. Characteristic Analysis of the Multistable Harvesters

The structural characteristics of some existing multistable-type piezoelectric harvesters are listed in Table 3. Magnetic attraction or magnetic repulsion can induce multistability. The structure could experience monostable, bistable, and tristable states by adjusting the separation and gap distances between magnets [163,164].

In addition to cantilever, the shape of piezoelectric beams can be of a composite shape. Zhang et al. proposed a tristable harvester that comprised a linear-arch composite beam with a tip-magnet attachment and two external magnets. The arched part of the composite beam influenced the response spectrum curve and the potential well depth of this system [167]. In addition to a one degree of freedom structure, a 2-DOF quadstable harvester was studied [171].

## 5. Magnetic Plucking Energy Harvesters

### 5.1. Working Mechanism

The magnetic force can be used as an intermediate driving force to drive the vibration of the piezoelectric beams, which convert the vibration energy into electrical energy [177,178,179,180,181,182,183,184,185,186,187,188,189]. It is better to harvest the energy of rotational motion. The magnetic plucking energy harvesters are divided into two groups based on the number of piezoelectric beams used in the harvesters.

For magnetic plucking energy harvesters, the piezoelectric cantilever is the same as that in the nonlinear energy harvester for vibration-to-electricity transduction. However, the difference between them is in how excitations are applied. In the nonlinear energy harvester, a piezoelectric cantilever and magnets as a unit is applied with base excitations. In magnetic plucking, first the piezoelectric cantilever stands still and is excited by magnetic force. The magnet, which follows motions from the external vibrations, interacts with another magnet fixed at the free end of the piezoelectric cantilever, and thus excites the cantilever to vibrate.

**Figure 13:**
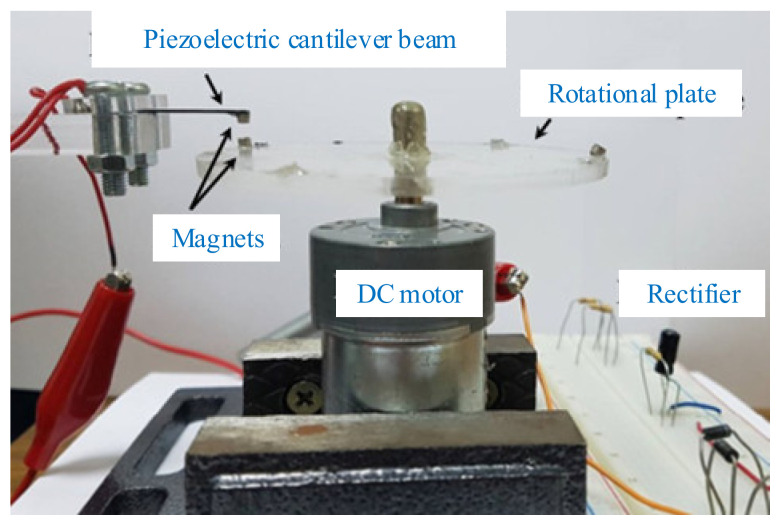
The rotational mechanism (reproduced with permission from [187]; published by Elsevier B.V., 2018).

Magnetic plucking is often applied to the strategy of frequency upconversion when energy sources, such as human motions, provide excitations with very low and irregular frequencies. Sensors can be powered by a wave energy harvester using such frequency-upconversion mechanism [57]. A slower-moving inertial mass magnetically plucks a piezoelectric cantilever beam, which converts mechanical energy to electrical energy at a higher frequency. Moreover, piezoelectric energy harvester excited by the magnetic force can be proposed and developed for rotational-mechanism applications. A noncontact magnetic force was applied to excite the piezoelectric cantilever vibration and avoid power frictional loss, as shown in Figure 13 [187].

### 5.2. Single Piezoelectric Beam

**Figure 14:**
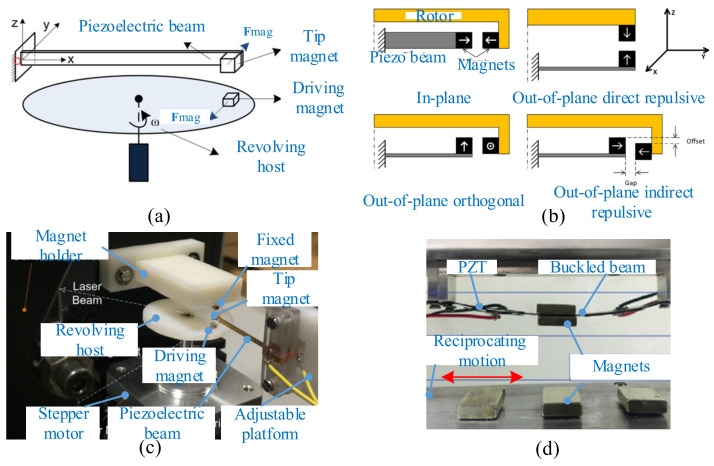
Magnetic plucking harvesters with a single piezoelectric beam. (**a**) The rotational energy harvester (reproduced with permission from [190]; published by Elsevier B.V., 2017). (**b**) Magnetic plucking configurations (reproduced with permission from [191]; published by Elsevier B.V., 2016). (**c**) A bistable frequency-upconverting harvester (reproduced with permission from [192]; published by Elsevier, 2018). (**d**) A multistep harvester (reproduced with permission from [193]; published by Elsevier, 2017).

The magnets were arranged on the rotating host and the cantilever beam. The angular kinetic energy was transferred to the vibrational energy of the piezoelectric beam via magnetic coupling between the magnets (Figure 14a). Fu and Yeatman investigated the arrangement of repulsion between magnets and the best was chosen for the merits of a high output power [190]. Frequency upconversion was achieved by magnetic plucking, and this harvester had a wide bandwidth at low rotational speeds. More than 20 μW of output power could achieve the rotational frequency ranging from 15 to 35 Hz. Xue and Roundy categorized several feasible magnet configurations to achieve magnetic plucking (Figure 14b). The piezoelectric beam could be deflected in or orthogonal to the plane of the rotating magnet motion with magnetic coupling. Results showed that a high-level optimization could be achieved by selection of the number and spacing of beams and magnets for both in-plane and out-of-plane plucking [191].

In order to enhance the power-generation capability over a wider bandwidth, Fu and Yeatman investigated a rotational energy harvester using frequency upconversion and bistability (Figure 14c). Another fixed magnet was mounted above the tip magnet at the free end of the beam. The repulsive magnetic force between this magnet and the tip magnet introduced two stable positions for the beam. Results showed that the bistable mechanism allowed the harvester to achieve effective energy harvesting at a low frequency when the harvester operated in the periodic double-well mode [192].

In addition to a piezoelectric cantilever, a clamped beam on both ends driven by a magnetic force can also be used for energy harvesting. Jiang et al. proposed a multistep buckled-beam piezoelectric energy harvester [193] (Figure 14d). The results showed that the magnet array with staggered magnetic poles increased the output voltage of this harvester by 25.0% compared to the unidirectional magnetic pole arrangement.

### 5.3. Multiple Piezoelectric Beams

**Figure 15:**
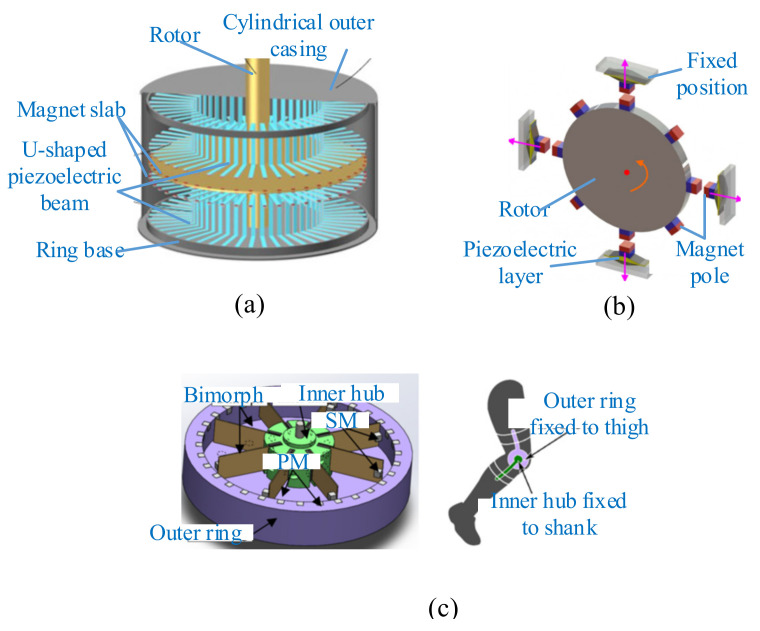
Magnetic plucking harvesters with multiple piezoelectric beams. (**a**) A U-shaped piezoelectric coupled harvester (reproduced with permission from [195]; published by Elsevier, 2019). (**b**) A rotational energy harvester (reproduced with permission from [196]; published by IOP, 2017). (**c**) A piezoelectric knee-joint harvester (reproduced with permission from [197]; published by IOP, 2016).

Multiple piezoelectric beams driven by magnetic force also were used in some studies for enhancing the overall generated power of the harvester. Ramezanpour et al. proposed a device consisting of a rotating proof mass and eight piezoelectric bimorph beams [194]. The magnet mounted on the rotating pendulum actuated the tips of the eight beams due to magnetic interaction. The results showed that the harvester with a relatively high angular velocity generated more voltage than that with a low angular velocity, and the generated voltage of the attractive case was more than the repulsive one. Adopting a U-shaped bimorph piezoelectric beam, Hu et al. presented a novel energy harvester that consisted of circularly distributed vibrators, a rotor, and a cylindrical outer casing [195] (Figure 15a). A comprehensive parameter analysis was conducted, including the rotor speed, the thicknesses of the substrate, and the piezoelectric patch. The results indicated that the substrate thickness had the greatest influence on the responses of this harvester. The electric power of this harvester could reach 8.19 kW under reasonable design. Zou et al. designed a novel magnetically coupled flextensional rotation energy harvester with buckled beams [196] (Figure 15b). As the excitation magnets rotated, periodically varying magnetic force was applied to the transducer with magnetic plucking. The component of the magnetic force perpendicular to the transducer was amplified and transmitted to the piezoelectric layer, and the voltage could be produced due to the piezoelectric effect. The results indicated that more excitation magnets and smaller excitation distance could significantly increase the harvested energy. A maximum power of 3.1 mW could be obtained with one flextensional transducer at a rotating speed of 1000 r/min.

In addition, the magnetic-plucking mechanism was also applied to harvest energy from human motion. Kuang et al. introduced a piezoelectric knee-joint energy harvester with frequency upconversion induced by magnetic plucking [197] (Figure 15c). During walking, the thigh and the shank rotated around the knee joint. Then the primary magnet (PM) actuated by the knee-joint motion excited the bimorphs through the secondary magnet (SM) fixed on the beam tip, and this device converted kinetic energy into electric power. An average power output of 5.8 mW could be acquired by knee-joint motion at a frequency of 0.9 Hz.

### 5.4. Characteristic Analysis of the Magnetic-Plucking Harvesters

Table 4 shows the performances of some existing magnetic-plucking-type piezoelectric harvesters. The magnetic-plucking configurations can be divided into two categories. One is the in-plane layout, where the piezoelectric beam is deflected in the plane of the driving magnet motion. The other is the out-of-plane, where the piezoelectric beam is deflected orthogonal to the plane of the driving magnet motion. The benefit of the out-of-plane layout is the possibility of placing multiple piezoelectric beams on a single substrate [191]. In addition, the magnetic force applied to piezoelectric beams is related to the magnetization direction, the position, and the rotation direction of the driving magnet. Furthermore, magnetic-plucking-type piezoelectric harvesters are mainly applied to frequency upconversion, which is suitable for harvesting in low-frequency and high-amplitude environments, such as in human motions. Pozzi designed a wearable knee-joint energy harvester that used magnetic plucking [179]. The rotation of the joint caused the relative rotation between the piezoelectric bimorphs holding permanent magnets and 65 ferromagnetic teeth. Under tests, this harvester generated electrical power of 50 mW and 70 mW from walking and running steps, respectively. Yeo et al. proposed a wrist-worn energy harvester using six trapezoidal-shaped cantilever beams with magnetic plucking [189]. The performance of this wrist-worn device was tested under pseudo walking on bench, wrist, and shaking in hand. Compared with a commercial self-powered watch on the same aluminum swing-arm, this harvester could generate power from low-frequency motion. As a whole, proper arrangement of driving magnets and piezoelectric beams will help to improve the performance of the magnetic-plucking piezoelectric energy harvesters.

## 6. Hybrid Piezoelectric–Electromagnetic Energy Harvesters

### 6.1. Working Mechanism

The induced electromotive force produced with a magnetic flux through coils varies according to Faraday’s law. To improve the energy-conversion efficiency and increase the power density of an energy harvester, the hybrid energy harvester using both piezoelectric and electromagnet conversion mechanisms has received great attention [198,199,200,201,202,203,204]. Hybrid piezoelectric–electromagnetic energy harvesters are divided into two groups, according to the number of electromagnetic units.

The hybrid energy harvester contains both piezoelectric and electromagnet conversions. Equation (2) can be expressed in Equations (8) and (9) [100]:

(8) $$\dot{y}+\frac{1}{RC_{p}\omega_{n}}\dot{y}=\dot{x}$$

(9) $$\dot{y}+\frac{R}{L\omega_{n}}\dot{y}=\dot{x}$$

where *Cp* is the capacitance of the piezoelectric element, *L* is the inductance of the harvesting coil, *ω_n_* is the short-circuit nominal frequency, and *y* is the electric quantity representing the induced voltage in piezoelectric harvesters and the induced current in electromagnetic ones. They are measured across an equivalent resistive load *R*.

**Figure 16:**
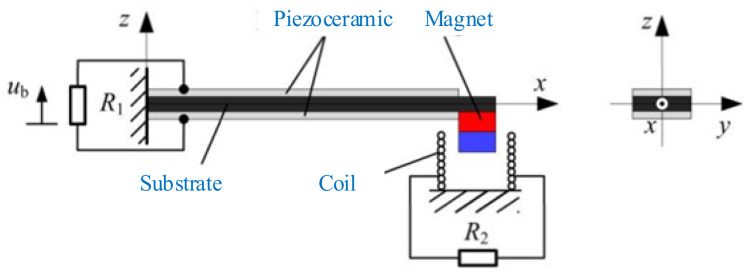
**A** typical hybrid energy harvester (reproduced with permission from [205]; published by MDPI, 2017).

**Figure 17:**
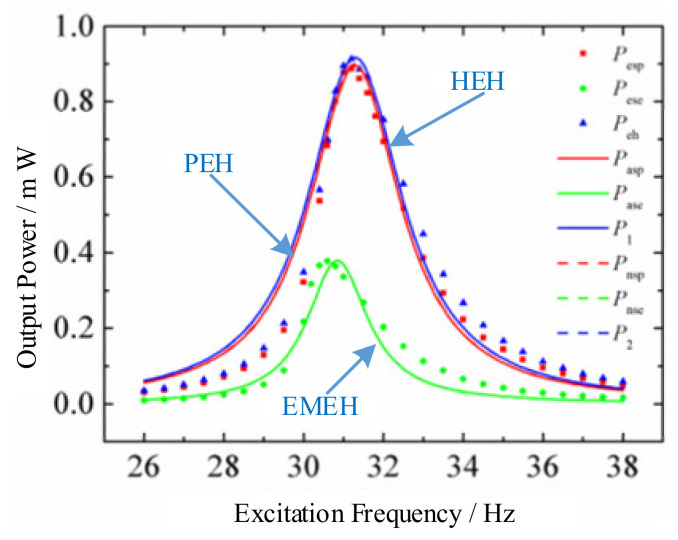
The optimal output power of the stand-alone piezoelectric energy harvester (PEH), stand-alone electromagnetic energy harvester (EMEH), and hybrid energy harvester (HEH) at different excitation frequencies (reproduced with permission from [205]; published by MDPI, 2017).

A typical hybrid energy harvester with the cantilever-beam structure is depicted in Figure 16 [205]. It comprises a piezoelectric cantilever beam with a permanent magnet as the tip mass and a cylindrical induction coil attached to the frame. The piezoelectric cantilever generates electricity by the piezoelectric effect. The magnet is used to tune the resonant frequency and amplify the deformation of the piezoelectric element. Meanwhile, the relative motion between the magnet and coil can induce electric current in the coil. The maximum output power of hybrid energy harvester (0.913 mW) with matched load resistances is 2.93% and 142.18% higher than the stand-alone piezoelectric energy harvester (0.887 mW) and electromagnetic energy harvester (0.377 mW), respectively. The operating frequency bandwidth of the hybrid energy harvester is 108% and 122.7% times wider than stand-alone piezoelectric and electromagnetic energy harvester, as shown in Figure 17. The hybrid energy harvester has a better computational efficiency.

### 6.2. One Electromagnetic Unit

**Figure 18:**
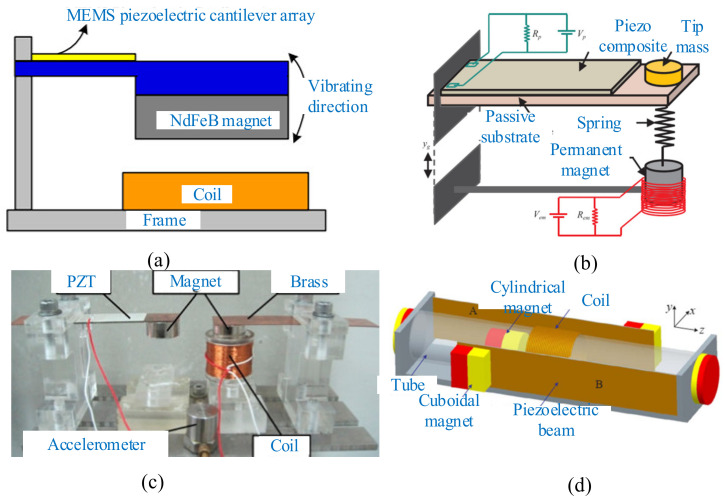
Representative designs with one electromagnetic unit. (**a**) A microvibration energy harvester (reproduced with permission from [208]; published by Elsevier B.V., 2014). (**b**) A 2-DOF system (reproduced with permission from [209]; published by Elsevier, 2017). (**c**) A multifrequency hybrid harvester (reproduced with permission from [210]; published by MDPI, 2019). (**d**) A bidirectional harvester (reproduced with permission from [211]; published by Elsevier, 2018).

The conventional hybrid harvester consists of a cantilever beam with a bonded piezoelectric patch and a permanent magnet attached to the tip, which oscillates within a coil fixed to the base [205,206,207]. Yu et al. developed a hybrid microvibration energy harvester that improved total output power and voltage in a low-frequency and small-amplitude vibration environment [208] (Figure 18a). Higher output voltage was obtained by using a piezoelectric cantilever array instead of a traditional single cantilever structure, and a low-power-consumption power-management circuit was designed. Experimental test results showed that the total maximum output power of this hybrid was 40.62 μW, with an optimal load resistance under the vibration acceleration of 0.2 g.

The drawback of the conventional model is that the power obtained is broad only over a certain region near a single resonant frequency, since it acts as a single degree of freedom system. Rajarathinam and Ali proposed a new model of hybrid energy harvester [209] (Figure 18b) comprising a permanent magnet attached to the free end of a cantilever beam with a linear elastic spring. The studies showed that the harvester operated over a broad range of frequencies as a 2-DOF system, compared to the conventional one. Xu et al. proposed three types of magnetically coupled hybrid energy harvesters that captured vibrational energy at two discrete frequencies. The superiority of this hybrid energy harvester was demonstrated [210] (Figure 18c). The device contained a typical nonlinear harvester with magnetically coupled dual beams. The hardening and softening nonlinear responses were observed at the first and second resonant frequencies through the frequency upward and downward sweeps, respectively. For this harvester, the optimal peak output powers (2.96 mW at 23.6 Hz and 4.76 mW at 32.8 Hz) were obtained under 2 m/s^2^ excitation acceleration.

Compared with the harvesters introduced in this part, Fan et al. proposed a novel hybrid harvester that had the capability of extracting energy from bidirectional excitations (*x* and *z*) [211] (Figure 18d). The piezoelectric part operated under the x-directional excitations. The center magnet moved along the tube’s axis and triggered the two piezoelectric beams to vibrate with the magnetic coupling in response to the z-directional excitation. Meanwhile, the relative motion between the center magnet and the coil also generated the power output.

### 6.3. Multiple Electromagnetic Units

**Figure 19:**
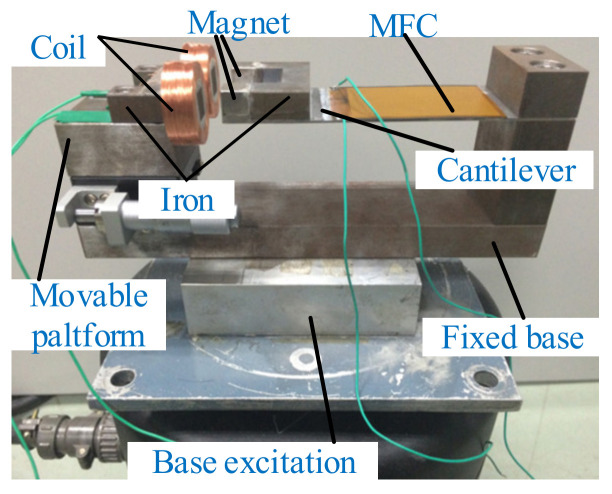
Representative designs with two electromagnetic units (reproduced with permission from [217]; published by Elsevier B.V., 2017).

Multiple electromagnetic units can be added to the hybrid piezoelectric and electromagnet system to improve the efficiency of energy harvesting [212,213,214,215]. Li et al. designed a nonlinear hybrid energy harvester that could tune the resonant frequency and improve the harvesting bandwidth at harmonic and random excitation [216]. Two coils were arranged on the upper and lower sides of the double-clamped beam. The resonant frequency and vibration response of this harvester could be tuned by changing the distance and polarity of the movable and fixed magnets. Xia et al. presented a tunable hybrid vibration energy harvester that included operating-frequency tuning and coupling-effect tuning [217] (Figure 19). Two coils were placed opposite to the magnets. The magnets and irons formed a well-designed closed magnetic circuit instead of the isolated magnet model to avoid unstable performance. The first modal frequency bandwidth, ranging from 25.5–62 Hz, was achieved at different relative areas between the magnets and the irons and at 2.78 mW average power over the frequency range obtained under a 0.3 g base acceleration. Benefiting from the proposed technique, the harvester could improve the environmental adaptability.

### 6.4. Characteristic Analysis of the Hybrid Harvesters

In general, a piezoelectric harvester usually generates higher voltage and lower current, but an electromagnetic harvester generates higher current and lower voltage [209]. The hybrid piezoelectric–electromagnetic harvester studied by scholars is expected to produce high voltage and high current simultaneously. Table 5 illustrates the performances of some typical hybrid piezoelectric–electromagnetic harvesters.

It can be seen in Table 5 that some research findings on hybrid harvester were linear and single-frequency. In this configuration, the harvesters generated the maximum power only under the resonant case. Therefore, some harvesters are designed for harvesting energy from broadband frequency vibrations. For example, the harvesters developed by Xu et al. [209] and Rajarathinam et al. [210] captured energy with two discrete frequencies. Moreover, some nonlinear technologies, such as frequency upconversion, magnetic tuning, and multistable can be applied to hybrid piezoelectric–electromagnetic harvester to enhance the energy harvesting performance. For example, Yang and Cao presented a novel tristable hybrid vibration energy harvester that was composed of a piezoelectric stack, two ring permanent magnets, and a central inertial mass based on a snap-through spring-mass system [203]. The piezoelectric-stack-generated energy undergoes vibrations. Meanwhile, the relative motions between the ring permanent magnets and the coil generated power due to electromagnetic induction. The hardening–softening spring effect of this tristable harvester produced a large output power, and it was suitable for collecting energy from low- and ultralow-level vibration resources.

## 7. Application

Piezoelectric vibration energy harvesting can also be applied in various areas, including buildings, MEMS, biomechanical and human motions, etc. [2,72,78,218,219]. This section focuses on the application of piezoelectric vibration energy harvesting techniques that adopt magnetic force.

**Figure 20:**
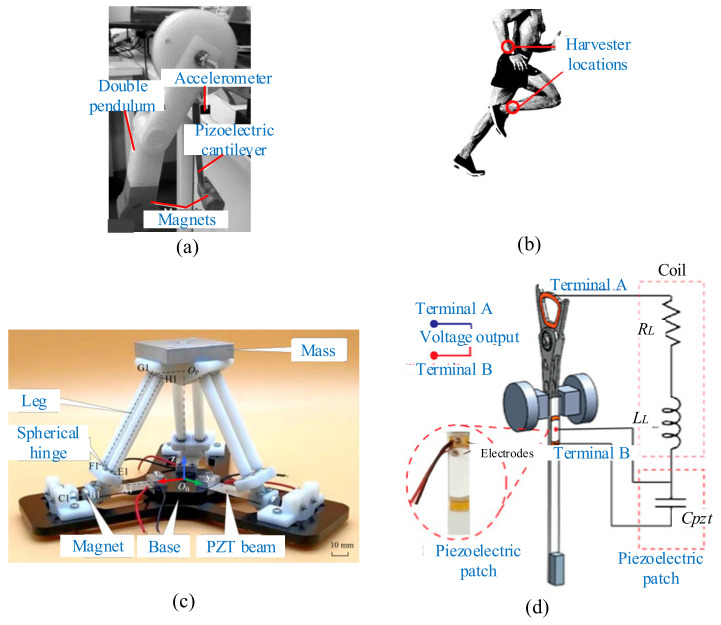
Representative design in applications. (**a**) A double-pendulum system (reproduced with permission from [223]; published by Elsevier, 2019). (**b**) Practical tests on a human leg and arm (reproduced with permission from [223]; published by Elsevier, 2019). (**c**) An arbitrary-directional harvester (reproduced with permission from [227]; published by IOP, 2019). (**d**) The hybrid harvester (reproduced with permission from [229]; published by MDPI, 2019).

One of the applications is powering implantable biomedical devices. Karami and Inman proposed energy harvesters to convert the vibrations from heartbeats to electrical energy for meeting the power requirement of a pacemaker [220]. Both monostable and bistable energy harvesters were designed according to the special signature of heart vibrations. This bistable device could generate power of more than 3 μW with the heart rate, which is about three times the power demand of the pacemaker. The batteries and the circuits of the pacemakers are encapsulated in a titanium case. Titanium is a biocompatible material. The sealed casing ensures that there is no contact between the interior of the body and the pacemaker batteries or circuits. Human motions such as arm or leg movements and breathing provide a particular energy source to power wearable and implantable devices [221,222]. Izadgoshasb et al. proposed an efficient piezoelectric harvester that extracted energy from human motions to replace a pacemaker battery [223] (Figure 20a). The use of magnetic coupling with a double-pendulum system was to improve the performance of this harvester. The prototype of this harvester was attached to a human leg and arm for practical tests (Figure 20b). The average power harvested from leg and arm motions could reach 86.12 μW and 76.25 μW in jogging modes, respectively.

In addition, another widespread application is in industry [224,225,226]. Yuan et al. explored a nonlinear arbitrary-directional broadband piezoelectric vibration energy harvester [227] (Figure 20c). This setup consisted of a 3-DOF parallel mechanism and three sets of polar-opposing permanent magnets. The proposed harvester could achieve effective energy in any direction with a wide bandwidth using the nonlinear magnetic force. The maximum average power of this harvester was 22.3 mW under an excitation acceleration of 1 g, which is about 2.2 times that of the linear one, and the bandwidth could be up to 5 Hz with a center frequency of 11 Hz. Yarar et al. investigated a MEMS-based energy-harvesting device [228]. The piezoelectric cantilever of this harvester was covered with aluminum scandiumnitride (AlScN) and driven by permanent magnets. The results showed that the magnetic plucking was more useful to use on a piezoelectric energy harvester for MEMS. The maximum peak power of 15.6 μW was obtained across a resistive load of 117210 Ω under an excitation acceleration of 1 g. Vargas and Tinoco presented a piezo-inductive device for energy harvesting [229] (Figure 20d). This configuration generated energy in two parts—the coil moving on the magnetic field, and a piezoelectric transducer. This hybrid harvester obtained higher electrical performance compared with the single mode.

For piezoelectric materials, PZT is used in many applications. However, its toxic nature may limit its widespread applications. The necessity of alternative-materials research is a challenge for materials scientists. Hybrid, lead-free, polymer-based piezocomposites can be efficiently utilized in energy harvesters. However, their piezoelectric performances still require further improvement for widespread use in biomedical devices. Despite the promising potential of implantable energy-harvesting devices, their in vivo application is limited by their cytotoxicity and long-term behavior in host organisms. More coherent and synergistic efforts are needed among material scientists and medical professionals [35,76,77].

## 8. Summary and Outlook

Piezoelectric vibration energy harvesting technology has attracted a lot of attention over the past decade, resulting in a wide variety of papers and applications. Piezoelectric energy harvesting with magnetic coupling promises a more meaningful solution to narrow bandwidth and low energy efficiency. First, this paper presented recent studies of piezoelectric vibration energy harvesting, especially the role of magnetic force in such harvesting. Then, the focus of this review, as summarized below, was on the current advances in effective piezoelectric vibration energy harvesting techniques with magnetic coupling, including monostable, bistable, multistable, magnetic plucking and hybrid piezoelectric–electromagnetic techniques. Finally, applications also were reviewed.

A nonlinear vibration energy harvester can be easily designed by using the magnetic force. Monostable, bistable, or multistable harvesters can be acquired by using magnetic attraction or repulsion. The motion state can be transformed around the monostable state, bistable state, and multistable state under a reasonable arrangement of magnets [50,230]. The monostable system exhibits hysteretic resonant behavior due to the nonlinear magnetic effect. When the system’s state becomes bistable, the frequency response curve becomes more complicated because of the intrawell, interwell, and chaos motions. The interwell motion is successful due to its remarkable advantages of large-amplitude and broadband characteristics. The magnetic-plucking harvester for rotational energy is simple and easy to realize frequency upconversion, because magnetic force designed as contact-free provides better realization. The number of piezoelectric transduction units, the excitation-magnet arrangement, and the excitation levels all have impacts on energy-harvesting efficiency. The hybrid scheme combining piezoelectric and electromagnetic energy-transduction units have been applied to generate electricity for low-power-requirement electronics. This scheme can significantly increase the harvested energy. It is possible that these methods of adopting magnetic force are very useful for designing efficient vibration-energy harvesters.

This paper provides a meaningful perspective for the design and application of the piezoelectric energy harvester with magnetic coupling. Further research work was carried out for developing new harvesters combining the merits of various methodologies to create a hybrid system for improving the adaptability of the harvester. Moreover, this magnetic-coupling technique can be used in a wide range of applications by harvesting various energies from wind, flow, sound, aeroelastic energy, etc. More effective storing circuits and new materials should be developed for achieving better performance in future research.

For piezoelectric vibration energy harvesting, in order to solve the problems of inadequate power density and narrow operating-frequency bandwidth, there have been variety of methods developed, including an array of coupled or uncoupled oscillators across various frequencies, frequency-tuning mechanisms through mechanical or electrical means, and various nonlinear vibratory phenomena. However, magnets are a popular choice to realize nonlinear methods. Monostable, bistable, multistable, frequency upconversion, internal resonance, frequency tuning and other dynamic behaviors can be obtained using magnets. Many nonlinear methods can be integrated to improve the performance of the system using magnets. However, there are disadvantages of the piezoelectric energy harvesters with magnetic coupling. For the hybrid piezoelectric–electromagnetic harvester, fabrication of electromagnetic part on micro- and nanoscales is an area of challenge because of the compact size and significant power rise with the input amplitude, especially with low-frequency vibrations. The magnetic fields can interfere with the use of equipment and may have an impact on the safety of users, especially in medical instruments. In the case of pacemakers, a magnetic field is usually avoided due to its effects on device operation. It should be considered whether a device is safe for a living organism.

Nonlinear vibration energy harvesting is still a continuously evolving field. Future research could involve the nonlinear coupling and integration of various nonlinear mechanisms. The ultimate goal for vibration-energy harvesting of maximum power output and broadband response remains an open challenge for ongoing and future research. There is still a gap between the current status and practical applications, especially in biomedical aspects. Another challenge may be in the preparation of piezoelectric scaffolds for use in bone regeneration, sensors for various stimuli, in vivo implantable devices, and e-skin [3,7,231,232]. More challenges exist and many problems remain to be solved in the area of piezoelectric vibration energy harvesting. Continued efforts will be necessary to formulate novel solutions to develop effective and practical energy harvesting devices.

## Figures and Tables

**Table 1:** Characteristics of some existing monostable-type piezoelectric harvesters.

Reference	Year	Piezoelectric	Base Layer	Magnet Arrangement	Magnetic	Nonlinear Effect
Upadrashta and Yang [109]	2016	MFC	Aluminium	Parallel	Attraction and repulsion	Combining softening and hardening
Krishnasamy et al. [107]	2018	MPC	Aluminium	Parallel	Attraction or repulsion	Antiresonance and internal resonance
Yang et al. [104]	2019	PVDF	SMSE	Parallel	Attraction	Softening
Cai and Harne [101]	2019	PZT-5 H	Laminated	Parallel	Repulsion	A trapezoidal beam and nonlinearity
Yang and Towfighian [103]	2019	M2807P2	Polymeric	Parallel or perpendicular	Repulsion	Combining softening and hardening
Chen et al. [112]	2016	PZT-5 A	Stainless steel	Parallel	Attraction	The L-shaped internal resonant harvester
Yang and Towfighian [114]	2017	PZT-5 A	Polymeric	Parallel	Repulsion	The two degrees of freedom internal resonant harvester
Zhang et al. [126]	2017	PZT	E = 206 GPa	Perpendicular	Repulsion	Dependence on initial free-end levitation position
Abdelmoula et al. [95]	2017	d_31_ = −170 pC/N	Aluminum	Perpendicular	Attraction	Softening or hardening
Rui et al. [97]	2018	MFC	Aluminum alloy	Perpendicular	Repulsion	Hardening
					Attraction	Softening
Fan et al. [125]	2018	PZT-5 H	Brass	Perpendicular	Attraction	Softening and stoppers
Shih and Su [123]	2019	M-2807-P2	E = 205 GPa	Perpendicular	Attraction and repulsion	In two orthogonal directions

**Table 2:** Characteristics of some existing bistable-type piezoelectric harvesters.

Reference	Year	Piezoelectric	Base Layer	Magnetic	Construction Features
Erturk and Inman [157]	2011	PZT-5 A	Tempered blue steel	Attraction	A ferromagnetic cantilever with two permanent magnets
Lan and Qin [158]	2017	PZT	E = 210 GPa	Attraction	The additional small magnet reduced the height of barrier between potential wells
Lu et al. [154]	2018	PZT-5 H	Carbon fiber laminate	Attraction	An E-shape broadband piezoelectric harvester
Ferrari et al. [143]	2010	PZT	Steel	Repulsive	A piezoelectric converter coupled to permanent magnets
Su et al. [150]	2013	PZT-5 H	Aluminum	Repulsive	Magnet-induced dual-cantilever
Zou et al. [135]	2016	PZT	Two raised metal layers	Repulsive	Bistable and flextensional mechanisms
Zou et al. [159]	2017	PZT	65 Mn	Repulsive	Used two inverted piezoelectric cantilever beams for rotational motion
Yang and Towfighian [146]	2017	*e*_31_ = −20 V∙m/N	EI = 0.003 Pa∙m^4^	Repulsive	A cantilever with a movable spring-magnet
Yao et al. [141]	2018	PZT	Brass	Repulsive	An L-shaped piezoelectric beam
Zhang et al. [142]	2018	PVDF	~	Repulsive	An arc-shaped piezoelectric cantilever
Li et al. [132]	2018	PZT-5 A	E = 205 GPa	Repulsive	A magnet-induced buckled piezoelectric energy harvester
Pan et al. [139]	2019	PZT-5 H	Steel	Repulsive	A buckling inverted piezoelectric beam

**Table 3:** Characteristics of some existing multistable-type piezoelectric harvesters.

Reference	Year	Piezoelectric	Base Layer	Multistable	Construction Features
Cao et al. [161]	2015	PZT-5A	Stainless steel	Tristable	The performances depended on the parameters h, θ, and d
Zhu et al. [164]	2017	e_31_ = 23 × 10^−10^ C/N	E = 205 GPa	Tristable	A tristable harvester using attractive magnets
Wang et al. [175]	2019	PZT	Stainless steel	Tristable	Improved magnetic-force model and mathematical model
Lai et al. [165]	2019	MFC	Beryllium bronze	Tristable	A multistable piezomagnetoelastic energy-harvester array
Zhang et al. [167]	2020	PVDF	~	Tristable	A tristable harvester with an arch composite beam
Zhou et al. [170]	2018	PZT-5 A	~	Quadstable	Shallower and wider potential wells
Abdelhameed et al. [171]	2019	K2512U1	Stainless steel	Quadstable	A broadband quadstable 2-DOF energy harvester
Kim and Seok [172]	2014	d_31_ = −23 pm/V	E = 210 GPa	Pentastable	Nonlinear magnetic attraction effect
Zhou et al. [174]	2017	e_31_ = 23 × 10^−10^ C/N	E = 205 GPa	Pentastable	Nonlinear magnetic force was generated by a tip magnet and four external permanent magnets on an inclined plane
Wang et al. [173]	2017	MFC	Steel	Pentastable	A piecewise-linear piezoelectric cantilever with magnetic coupling

**Table 4:** Performances of some existing magnetic-plucking-type piezoelectric harvesters.

Reference	Year	Piezoelectric	Piezoelectric Beams	Magnetic	Magnet Configurations	Excitation Frequency (Hz)	Resistive Load (kΩ)	Beam Size L × B × H (mm^3^)	Power (μW)	Nonlinear Effect
Fu and Yeatman [190]	2017	e_31_ = −22.2 V·m/N	One	Repulsion	In-plane	15–35	100	26.6 × 1.5 × 0.1	20	Frequency upconversion
Xue and Roundy [191]	2017	PSI–5A4E	One	Repulsion	In-plane	5	120	26.4 × 3.2 × 0.1	3200	Frequency upconversion
Jiang et al. [193]	2017	PZT-5 H	One	Attraction and Repulsion	Out-of-plane	3.0	3300	~	5.0	Multistep mechanism and bistability
Fu and Yeatman [192]	2019	e_31_ = −22.2 V∙m/N	One	Repulsion	In-plane	10	150	33.5 × 2 × 0.1	52	Bistability and frequency upconversion
Ramezanpour et al. [194]	2015	PZT	Eight	Attraction	Out-of-plane	1.67	980.5	55.01 × 6.2 × 0.23	About 140	Frequency upconversion
Kuang et al. [197]	2016	PZT	Eight	Repulsion	In-plane	0.9	15	38.1 × 12.7 × 0.38	5800	Frequency upconversion
Zou et al. [196]	2017	PZT	~	Repulsion	Out-of-plane	17	240	40 × 10 × 1	220 (one)	Magnetically coupled flextensional harvester
Yeo et al. [189]	2018	PZT	Six	Repulsion	Out-of-plane	1	18	V = 1.54 × 10^−2^	42	Frequency upconversion

**Table 5:** Performances of some reported hybrid piezoelectric–electromagnetic harvesters.

Reference	Year	Piezoelectric	EM Unit	Acc. (m/s^2^)	Frequency (Hz)	PE Load (kΩ)	EM Load (Ω)	Beam Size L × B × H (mm^3^)	Power (mW)	Construction Features
Yu et al. [208]	2015	PZT	one	0.2 g	55.9	300	400	5 × 2.4 × 0.05	0.040.62 (total)	MEMS
Salim et al. [207]	2016	PZT	one	0.25 g	36	300	10	25 × 12.7 × 0.13	0.710 (total)	Ring magnets with a hanging coil inside
Sriramdas et al. [206]	2018	PZT	one	0.5 g	22.8	286	775	90 × 3.5 × 0.3	1.7 (total)	A coil oscillating inside a magnetic field
Rajarathinam and Ali [209]	2018	MFC	one	~	2.1 and 14.2	50	60	160 × 32 × 1	~	Nonlinear and 2-DOF
Fan et al. [211]	2018	PZT-5H	one	1.5 g	~	180	9	33 × 7 × 0.1	1.23 (EM) and 0.18 (PE)	Frequency upconversion and bidirectional
Xu et al. [210]	2019	P-5H	one	2	23.6 and 32.8	130	2200	60 × 20 × 0.5	2.96 and 4.76 (total)	Multifrequency
Xia et al. [217]	2017	MFC	two	0.3 g	~	~	~	V = 712 (PE) + 1000 (EM)	3.32 (total)	Magnetic tuning technique
Li et al. [216]	2017	PZT	two	0.2 g	~	152.5	17.3	20 × 8 × 3.8	0.46 (total)	Tuning of the resonant frequency and improvment of the harvesting bandwidth

## Data Availability

The data in the paper is in line with MDPI Research Data Policies.
